# Supplementary material for: Aprotic Ionic Liquids: A Framework for Predicting Vaporization Thermodynamics
Source: Molecules. 2022 Apr 3;27(7):2321. doi: 10.3390/molecules27072321 (PMC9000287; doi:10.3390/molecules27072321)
Supplement: Supplementary file 1 [file molecules-27-02321-s001.zip › molecules-1636436-supplementary.pdf]

Supporting information

Molecules, 2022

Aprotic Ionic Liquids: A Framework for Predicting Vaporiza-tion Thermodynamics

Sergey P. Verevkin,[a,b,*] Dzmitry H. Zaitsau,[a,b] Ralf Ludwig[a,b,c]

[a] Institut für Chemie, Abteilung für Physikalische Chemie, Universität Rostock, 18059, Rostock, Germany
[b] Department Life,Light&Matter, University of Rostock, 18059 Rostock, Germany
[c] Leibniz-Institut für Katalyse an der Universität Rostock *e.V.*, 18059 Rostock, Germany

Table S1
Provenance and purity of ILs used in this work.

IL [a]	CAS	source	purity [b]
[1-C_8-Py][NTf_2]	384347-06-2	Sigma-Aldrich	≥0.99
[3-Me-1-C_3-Py][NTf_2]	817575-06-7	Sigma-Aldrich	≥0.99
[3-CN-1-C_6-Py][NTf_2]	1447958-51-1	Sigma-Aldrich	≥0.99
[4-CN-1-C_6-Py][NTf_2]	956592-35-1	Sigma-Aldrich	≥0.99
[3-CN-1-C_8-Py][NTf_2]	1447958-52-2	Sigma-Aldrich	≥0.99

[a] The abbreviation [NTf_2] = bis(trifluoromethylsulfonyl)imide.
[b] Purity stated by the supplier from HPLC analysis

Quartz Crystal Microbalance (QCM)

Vapor pressures and molar enthalpies of vaporization of ILs, were measured by using the QCM method [1]. A sample of an IL was placed in an open cavity (Langmuir evaporation) inside of the thermostat block and it was exposed to vacuum (10^{-5} Pa) with the whole open surface of the loaded compound. The QCM-sensor was mounted directly above the measuring cavity containing the sample. Along the vaporization into high vacuum, a certain amount of sample was condensed on the quartz crystal surface. The change of the vibrational frequency Δf was recorded. It is directly related to the mass deposition Δm on the QCM according to the Sauerbrey equation [2]:

$$\Delta f = -\,C \times f^{2} \times \Delta m \times S_{C}^{-1} \quad \text{(S1)}$$

where f is the fundamental frequency of the crystal (6 MHz in this case) with $\Delta f << f$, S_C is the surface of the crystal, and C is a constant [2]. The measured frequency change rates ($df/d\tau$) can be used for calculation of absolute vapor pressures p_s according to equation:

$$p_s = K' \frac{df}{dt}\sqrt{\frac{T}{M}}. \quad \text{(S2)}$$

where the $K' = (9.5 \pm 1.1) \cdot 10^{-6}$ Pa·s·kg$^{1/2}$·Hz^{-1}·K$^{-1/2}$·mol$^{-1/2}$ [3] is the empirical calibration constant including all parameters involved in Eq. 1, as well as the all apparatus geometry specific parameters. Calibration of the set up was performed with the help of reliable vapor pressure data on [C_nPy][NTf_2], [$C_nC_n$im][NTf_2], and [$C_n$mim][NTf_2] series of ionic liquids. Standard molar enthalpy of vaporization, $\Delta_l^g H_m^o(T_0)$, was calculated as follows:

$$\ln\left(\frac{df}{dt}\sqrt{T}\right)=A'-\frac{\Delta_l^g H_m^o(T_0)-\Delta_l^g C_{p_m}^o T_0}{R}\left(\frac{1}{T}-\frac{1}{T_0}\right)+\frac{\Delta_l^g C_{p_m}^o}{R}\ln\left(\frac{T}{T_0}\right) \qquad \text{(S3)}$$

where T_0 is an arbitrarily chosen reference temperature (T_0 average temperature of experimental range of the QCM study) and A' is the empirical constant. The value $\Delta_l^g C_{p,m}^o = C_{p,m}^o(g) - C_{p,m}^o(l)$ is the difference between the molar heat capacities of the gaseous $C_{p,m}^o(g)$ and the liquid phase $C_{p,m}^o(l)$ respectively. A typical experiment was performed in a few consequent series with increasing and decreasing temperature steps. Every series included from 7 to 11 temperature points of mass loss rate determination. Such a procedure allowed for detection of any possible decomposition during frequency loss rate ($df/d\tau$) measurements. Reproducibility of results was established in series of randomly performed experimental runs. The study was considered as completed when the $\Delta_l^g H_m^o(T)$-values derived from the temperature-dependent rates ($df/d\tau$) achieved the level of uncertainty of ± 1 kJ·mol^{-1}. Primary experimental results of the QCM studies are provided in Table S1. The absence of decomposition of IL under experimental conditions was controlled by using spectroscopy. The residual amount of IL in the cavity, as well as the IL-deposit on QCM were analyzed by ATR-IR spectroscopy. No changes in the spectra have been detected, as can be seen in Figures S1 to S5 of the Supporting Information.

Table S2

Results of vapor pressures temperature dependence and vaporization enthalpies $\Delta_l^g H_m^o(T)$ measured using the QCM.[a]

Run	T / K	$df \cdot d\tau^{-1}$ / Hz·s^{-1}	$10^6 \cdot p_i$/ Pa	T^{-1} / K^{-1}	$R \cdot \ln(p_i/p^o)$	$\frac{\Delta_l^g H_m^o(T)}{\text{kJ·mol}^{-1}}$
[1-C8-Py][NTf2]						
$\ln(p_i\ /p^o) = -\frac{76539}{RT_0} - \frac{142439}{R}\left(\frac{1}{T}-\frac{1}{T_0}\right) - \frac{85}{R}\left(\frac{T_0}{T} - 1 - \ln\left(\frac{T}{T_0}\right)\right)$, T_0 = 406.2						
1	427.91	0.4213	120	0.002337	-170.8	140.6
	422.97	0.2655	75	0.002364	-174.6	141.0
	418.06	0.1643	46	0.002392	-178.7	141.4
	413.08	0.1009	28	0.002421	-182.8	141.9
	408.12	0.0624	17	0.002450	-186.8	142.3
	403.13	0.03743	10	0.002481	-191.1	142.7
	398.14	0.02211	6.1	0.002512	-195.6	143.1
	393.14	0.01264	3.5	0.002544	-200.3	143.5
	388.15	0.00718	2.0	0.002576	-205.0	144.0
	383.15	0.004132	1.1	0.002610	-209.7	144.4
2	430.44	0.5360	154	0.002323	-168.7	140.4
	425.42	0.3345	95	0.002351	-172.7	140.8
	420.40	0.2086	59	0.002379	-176.7	141.2
	415.37	0.12972	37	0.002407	-180.7	141.7
	410.35	0.07965	22	0.002437	-184.8	142.1
	405.33	0.04847	13	0.002467	-189.0	142.5
	400.31	0.02844	7.9	0.002498	-193.4	142.9
	395.30	0.01689	4.6	0.002530	-197.8	143.4
	390.29	0.009287	2.5	0.002562	-202.9	143.8
	385.30	0.005300	1.4	0.002595	-207.6	144.2
[3-Me-1-C3-Py][NTf2]						
$\ln(p_i\ /p^o) = -\frac{71721}{RT_0} - \frac{126207}{R}\left(\frac{1}{T}-\frac{1}{T_0}\right) - \frac{70}{R}\left(\frac{T_0}{T} - 1 - \ln\left(\frac{T}{T_0}\right)\right)$, T_0 = 386.3 K						

	407.75	0.5543	170	0.002452	-167.9	126.2
	402.85	0.3524	107	0.002482	-171.7	126.2
	397.98	0.2223	67	0.002513	-175.6	126.2
	393.02	0.1372	41	0.002544	-179.7	126.2
	388.05	0.08464	25	0.002577	-183.7	126.2
	383.07	0.05092	15	0.002610	-188.0	126.2
1	378.10	0.03047	9.0	0.002645	-192.3	126.2
	373.12	0.01782	5.2	0.002680	-196.9	126.2
	405.33	0.4339	132	0.002467	-170.0	126.2
	400.30	0.2704	82	0.002498	-173.9	126.2
	395.28	0.1681	51	0.002530	-178.0	126.2
	390.27	0.1047	31	0.002562	-181.9	126.2
	385.26	0.06349	19	0.002596	-186.2	126.2
	405.24	0.4257	130	0.002468	-170.1	124.2
	400.20	0.2669	81	0.002499	-174.1	124.5
	395.17	0.1656	50	0.002531	-178.1	124.9
	390.14	0.1011	30	0.002563	-182.2	125.2
	385.13	0.06203	18	0.002597	-186.3	125.6
2	380.12	0.03727	11	0.002631	-190.6	125.9
	375.11	0.02207	6.5	0.002666	-195.1	126.3
	370.11	0.01305	3.8	0.002702	-199.5	126.6
	365.12	0.007481	2.2	0.002739	-204.2	127.0
	360.13	0.004229	1.2	0.002777	-209.0	127.3
	402.68	0.3426	104	0.002483	-172.0	124.3
	397.63	0.2119	64	0.002515	-176.0	124.7
	392.60	0.1296	39	0.002547	-180.1	125.0
	402.74	0.35581	108	0.002483	-171.6	124.3
	397.74	0.2225	67	0.002514	-175.6	124.7
	392.78	0.1380	41	0.002546	-179.6	125.0
3	387.81	0.0844	25	0.002579	-183.8	125.4
	382.84	0.05063	15	0.002612	-188.1	125.7
	377.86	0.02997	8.8	0.002646	-192.5	126.1
	372.89	0.01759	5.2	0.002682	-197.0	126.4
	367.92	0.01039	3.0	0.002718	-201.4	126.8
	362.93	0.005866	1.7	0.002755	-206.2	127.1
	357.94	0.003199	0.92	0.002794	-211.3	127.5

[3-CN-1-C_4-Py][NTf_2]

$$\ln(p_i/p^{o}) = -\frac{78549}{RT_0} - \frac{141366}{R}\left(\frac{1}{T} - \frac{1}{T_0}\right) - \frac{73}{R}\left(\frac{T_0}{T} - 1 - \ln\left(\frac{T}{T_0}\right)\right),\ T_0 = 424.8\ \text{K}$$

	448.10	0.5837	182	0.002232	-167.3	139.7
	443.07	0.3731	115	0.002257	-171.1	140.0
	438.07	0.2394	74	0.002283	-174.8	140.4
	433.06	0.1508	46	0.002309	-178.7	140.8
1	428.03	0.09822	30	0.002336	-182.3	141.1
	423.03	0.06036	18	0.002364	-186.4	141.5
	418.01	0.03753	11	0.002392	-190.4	141.9
	413.02	0.02322	6.9	0.002421	-194.5	142.2
	407.99	0.01401	4.2	0.002451	-198.7	142.6
	402.97	0.008292	2.4	0.002482	-203.1	143.0

2	445.56	0.4524	140	0.002244	-169.5	139.9
	440.53	0.2902	90	0.002270	-173.2	140.2
	435.44	0.1866	57	0.002297	-176.9	140.6
	430.42	0.1192	36	0.002323	-180.7	141.0
	425.40	0.07502	23	0.002351	-184.6	141.3
	420.38	0.04756	14	0.002379	-188.5	141.7
	415.37	0.03000	9.0	0.002408	-192.3	142.1
	410.36	0.01809	5.4	0.002437	-196.6	142.4
	405.33	0.01090	3.2	0.002467	-200.9	142.8
	400.32	0.006528	1.9	0.002498	-205.2	143.2
[3-CN-1-C_6-Py][NTf_2]						
$\ln(p_i\ /p^\circ) = -\frac{79609}{RT_0} - \frac{151076}{R}\left(\frac{1}{T} - \frac{1}{T_0}\right) - \frac{81}{R}\left(\frac{T_0}{T} - 1 - \ln\left(\frac{T}{T_0}\right)\right)$, T_0 = 426.2 K						
1	448.02	0.4775	140	0.002232	-169.5	149.3
	443.02	0.3018	88	0.002257	-173.4	149.7
	437.97	0.1899	55	0.002283	-177.3	150.1
	432.95	0.1176	34	0.002310	-181.3	150.5
	427.92	0.0720	21	0.002337	-185.4	150.9
	422.91	0.04424	13	0.002365	-189.5	151.3
	417.90	0.02596	7.4	0.002393	-194.0	151.7
	412.89	0.01529	4.3	0.002422	-198.4	152.2
	407.89	0.00915	2.6	0.002452	-202.8	152.6
	402.88	0.005151	1.4	0.002482	-207.6	153.0
2	450.62	0.5887	173	0.002219	-167.7	149.1
	445.57	0.3722	109	0.002244	-171.6	149.5
	440.58	0.2380	69	0.002270	-175.4	149.9
	435.56	0.14871	43	0.002296	-179.3	150.3
	430.54	0.09282	27	0.002323	-183.3	150.7
	425.53	0.05809	17	0.002350	-187.2	151.1
	420.51	0.03458	9.8	0.002378	-191.6	151.5
	415.50	0.02058	5.8	0.002407	-196.0	151.9
	410.50	0.012229	3.4	0.002436	-200.3	152.3
	405.50	0.007189	2.0	0.002466	-204.8	152.8
[4-CN-1-C_6-Py][NTf_2]						
$\ln(p_i\ /p^\circ) = -\frac{78270}{RT_0} - \frac{147872}{R}\left(\frac{1}{T} - \frac{1}{T_0}\right) - \frac{81}{R}\left(\frac{T_0}{T} - 1 - \ln\left(\frac{T}{T_0}\right)\right)$, T_0 = 427.5 K						
1	445.52	0.4982	146	0.002245	-169.2	146.4
	440.48	0.3145	92	0.002270	-173.0	146.8
	435.44	0.2003	58	0.002297	-176.8	147.2
	430.43	0.1273	37	0.002323	-180.7	147.6
	425.42	0.07928	23	0.002351	-184.6	148.0
	420.40	0.04757	14	0.002379	-188.9	148.4
	415.40	0.02880	8.1	0.002407	-193.2	148.9
	410.39	0.01705	4.8	0.002437	-197.6	149.3
	405.38	0.009962	2.8	0.002467	-202.1	149.7
2	448.08	0.6316	185	0.002232	-167.2	146.2
	443.08	0.3995	117	0.002257	-171.0	146.6
	438.06	0.2555	74	0.002283	-174.8	147.0
	433.05	0.1617	47	0.002309	-178.6	147.4

	428.06	0.1000	29	0.002336	-182.7	147.8
	423.05	0.06228	18	0.002364	-186.7	148.2
	418.03	0.03794	11	0.002392	-190.8	148.6
	413.05	0.02212	6.2	0.002421	-195.4	149.0
[3-CN-1-C_8-Py][NTf_2]						
$\ln(p_i/p^o) = -\frac{79694}{RT_0} - \frac{152939}{R}\left(\frac{1}{T}-\frac{1}{T_0}\right) - \frac{89}{R}\left(\frac{T_0}{T} - 1 - \ln\left(\frac{T}{T_0}\right)\right)$, T_0 = 434.8 K						
1	453.10	0.5040	149	0.002207	-169.0	151.3
1	448.08	0.3151	92	0.002232	-173.0	151.8
1	443.07	0.1959	57	0.002257	-177.0	152.2
1	438.01	0.1245	36	0.002283	-180.8	152.7
1	432.99	0.07641	22	0.002310	-184.9	153.1
1	427.97	0.04718	14	0.002337	-188.9	153.5
1	422.97	0.02837	8.1	0.002364	-193.2	154.0
1	455.66	0.6279	186	0.002195	-167.2	151.1
1	450.59	0.3986	117	0.002219	-171.0	151.5
2	445.58	0.2504	73	0.002244	-174.9	152.0
2	440.57	0.1598	46	0.002270	-178.7	152.4
2	435.55	0.1012	29	0.002296	-182.5	152.9
2	430.53	0.06098	18	0.002323	-186.8	153.3
2	425.52	0.03692	11	0.002350	-191.0	153.8
2	420.51	0.02210	6.3	0.002378	-195.3	154.2
2	415.50	0.01326	3.7	0.002407	-199.6	154.7
2	453.05	0.5015	148	0.002207	-169.0	151.3
3	448.06	0.3188	94	0.002232	-172.9	151.8
3	443.02	0.1992	58	0.002257	-176.8	152.2
3	437.99	0.1237	36	0.002283	-180.8	152.7
3	432.98	0.07552	22	0.002310	-185.0	153.1
3	427.95	0.04677	13	0.002337	-189.0	153.5
3	422.93	0.02821	8.0	0.002364	-193.3	154.0
3	417.91	0.01686	4.8	0.002393	-197.6	154.4
3	412.90	0.009843	2.8	0.002422	-202.1	154.9
3	407.90	0.005781	1.6	0.002452	-206.6	155.3
4	455.62	0.6266	185	0.002195	-167.2	151.1
4	450.59	0.3952	116	0.002219	-171.0	151.5
4	445.58	0.2523	74	0.002244	-174.8	152.0
4	440.57	0.1575	46	0.002270	-178.8	152.4
4	435.55	0.09851	29	0.002296	-182.7	152.9
4	430.52	0.06197	18	0.002323	-186.6	153.3
4	425.50	0.03790	11	0.002350	-190.8	153.8
4	420.49	0.02282	6.5	0.002378	-195.0	154.2

[a] Combined expanded uncertainties are U_c(T) = 0.02 K, $U_r(df\cdot dt^{-1})$ = 0.01 $U_r(p_{sat})$ = 0.5 for confidence level = 0.95, $k \approx 2$.

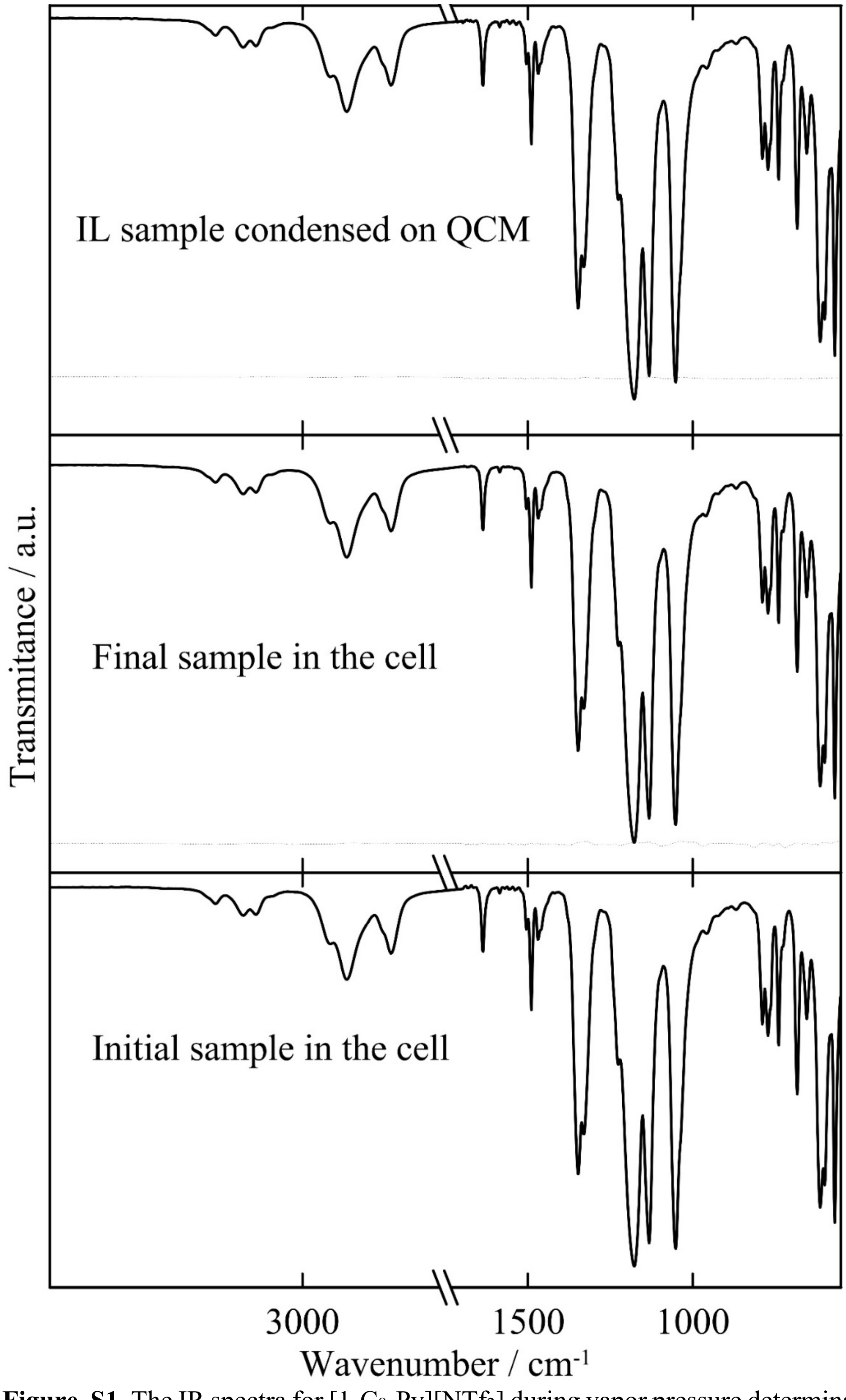

Figure. S1. The IR spectra for [1-C_8-Py][NTf_2] during vapor pressure determination with QCM method. Dotted line corresponds to the difference between presented spectrum and spectrum of the initial sample. All deviations in spectra stems from small amount of sample condensed at the surface of QCM.

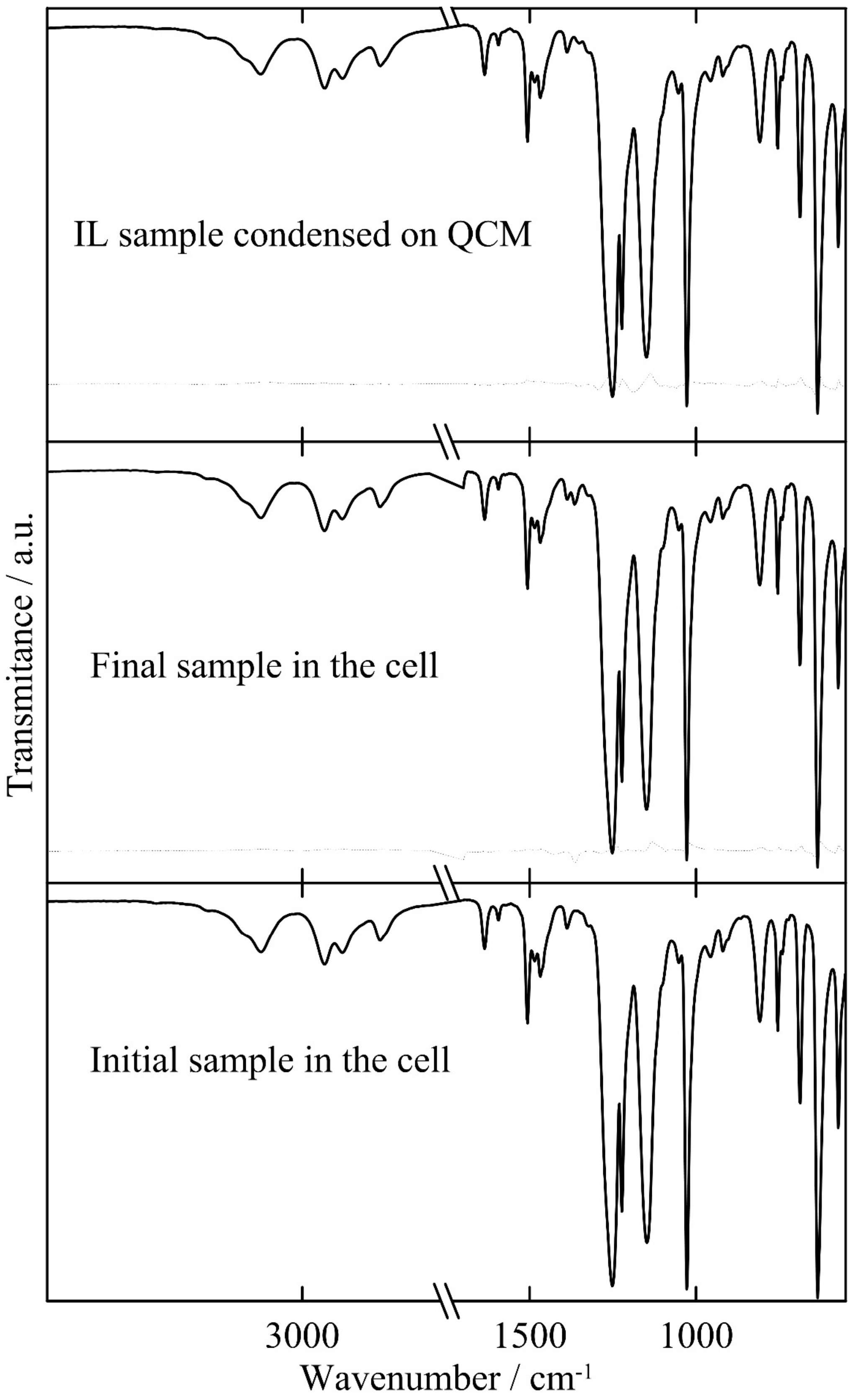

Figure. S2. The IR spectra for [3-Me-1-C_4-Py][NTf_2] during vapor pressure determination with QCM method. Dotted line corresponds to the difference between presented spectrum and spectrum of the initial sample. All deviations in spectra stems from small amount of sample condensed at the surface of QCM.

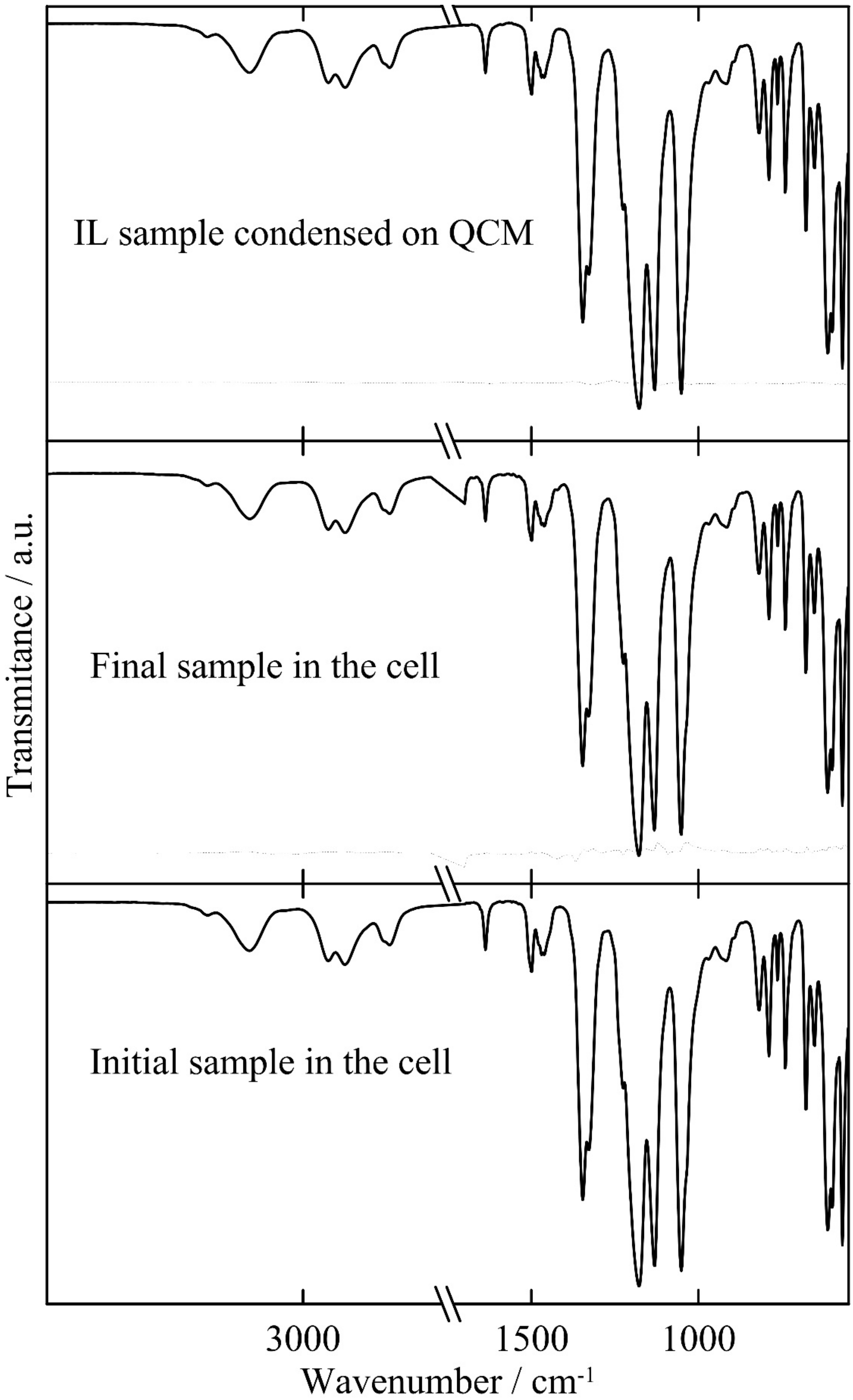

Figure. S3. The IR spectra for [3-CN-1-C_6-Py][NTf_2] during vapor pressure determination with QCM. Dotted line corresponds to the difference between presented spectrum and spectrum of the initial sample. All deviations in spectra stems from small amount of sample condensed at the surface of QCM.

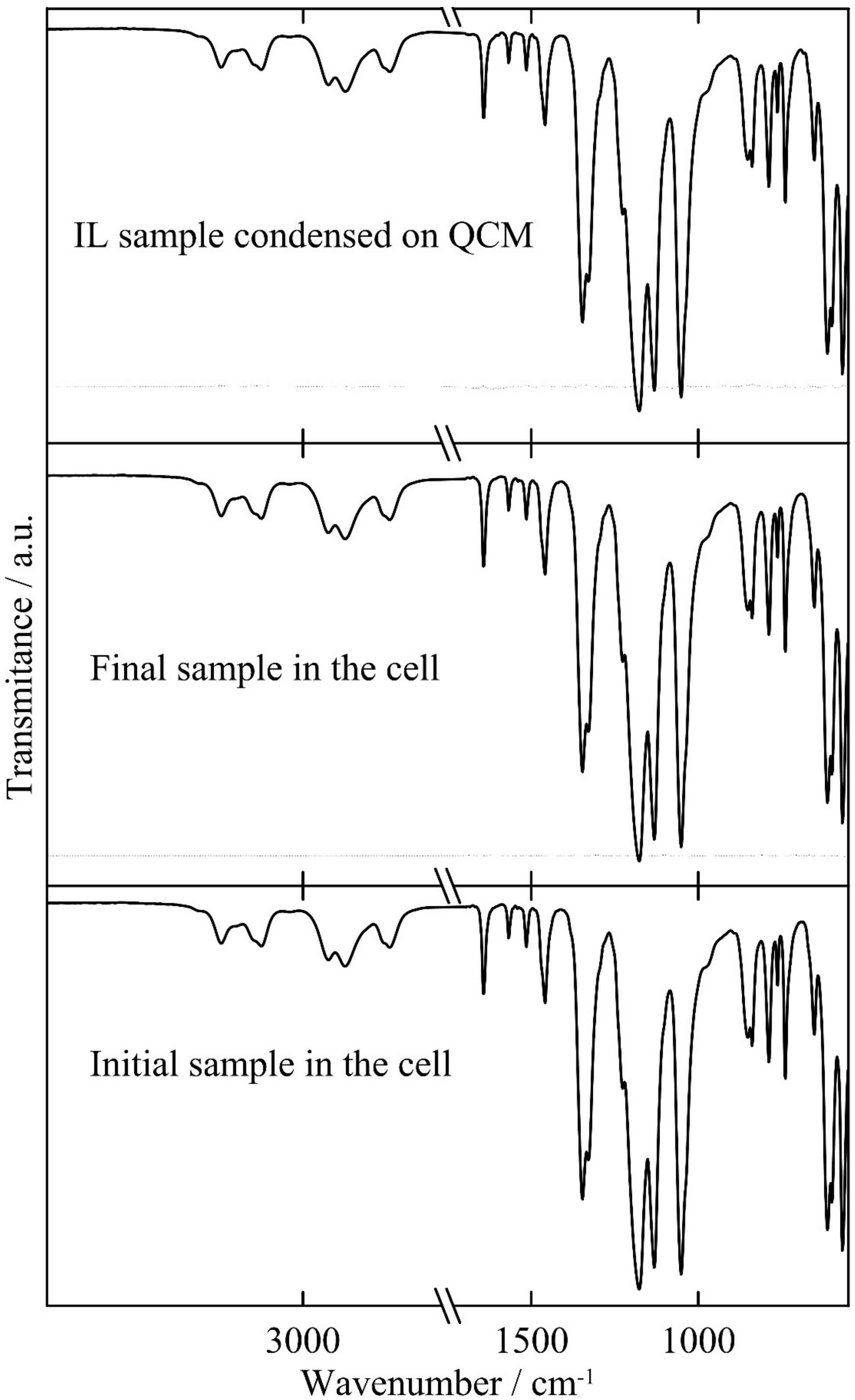

Figure. S4. The IR spectra for [4-CN-1-C_6-Py][NTf_2] during vapor pressure determination with QCM method. Dotted line corresponds to the difference between presented spectrum and spectrum of the initial sample. All deviations in spectra stems from small amount of sample condensed at the surface of QCM.

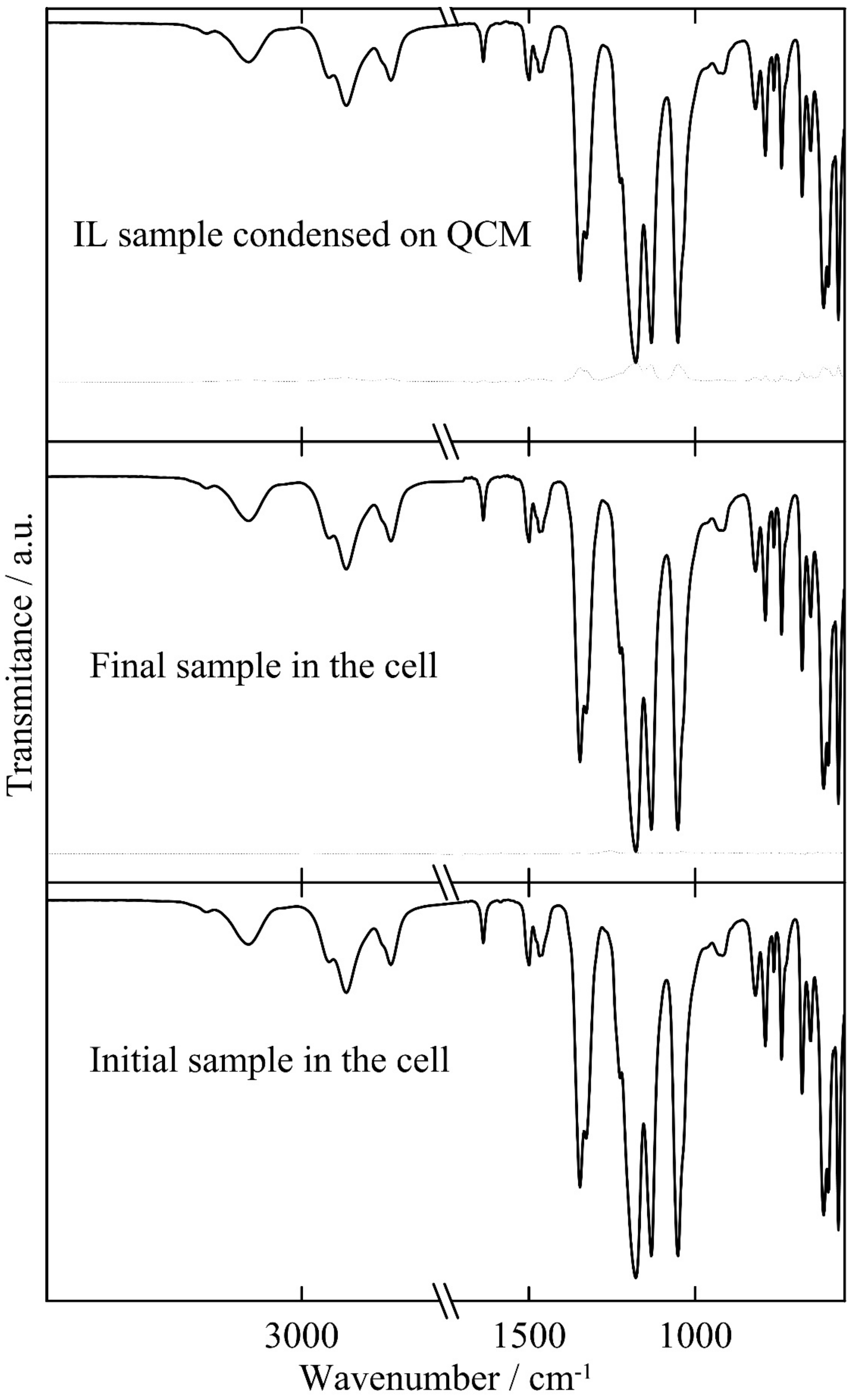

Figure. S5. The IR spectra for [3-CN-1-C_8-Py][NTf_2] during vapor pressure determination with QCM method. Dotted line corresponds to the difference between presented spectrum and spectrum of the initial sample. All deviations in spectra stems from small amount of sample condensed at the surface of QCM.

Table S3

Compilation of data on molar heat capacities, $C^{o}_{p,m}$, and heat capacity differences $\Delta^{g}_{l}C^{o}_{p,m}$ (in J·K^{-1}·mol^{-1}) at T = 298.15 K for pyridinium based ionic liquids.

IL	N	$C^{o}_{p,m}(liq)_{exp}$	$C^{o}_{p,m}(liq)_{calc}$ [a]	Δ[b]	$\Delta^{g}_{l}C^{o}_{p,m}$
[1-C_2-Py][NTf_2]	8	478.6 [4]	478.6	0	-62
[1-C_3-Py][NTf_2]	9	503.4 [4]	512	-9	-65
[1-C_4-Py][NTf_2]	10	540.2 [4]	546	-6	-70
[1-C_6-Py][NTf_2]	12	599.5 [4]	613	-13	-77
[1-C_8-Py][NTf_2]	14	662.8 [4]	679	-17	-85
[3-CN-1-C_4-Py][NTf_2]	11	586 [5]	579	7	-75
[3-CN-1-C_6-Py][NTf_2]	13	658 [5]	646	12	-84
[4-CN-1-C_6-Py][NTf_2]	13	633 [5]	646	-13	-81
[3-CN-1-C_8-Py][NTf_2]	15	709 [5]	713	-4	-91
[2-Et-1-C_2-Py][NTf_2]	10	(566.1) [6]	546	20	-70
[2-Et-1-C_3-Py][NTf_2]	11	593.9 [6]	579	15	-76
[2-Et-1-C_4-Py][NTf_2]	12	623.6 [6]	613	11	-80
[2-Et-1-C_5-Py][NTf_2]	13	652.7 [6]	646	7	-84
[2-Et-1-C_6-Py][NTf_2]	14	685.5 [6]	679	6	-88
[2-Et-1-C_7-Py][NTf_2]	15	717.5 [6]	713	5	-92
[2-Et-1-C_8-Py][NTf_2]	16	749.4 [6]	746	3	-96
[2-Et-1-C_9-Py][NTf_2]	17	778.5 [6]	780	-1	-100
[2-Et-1-C_{10}-Py][NTf_2]	18	811.2 [6]	813	-2	-104

[a] Calculated according to the empirical equation:

$C^{o}_{p,m}(liq)_{calc}$/J·K^{-1}·mol^{-1} = 33.4×N_{C+N} +211.7 with (R^2 = 0.9897), where N_{C+N} = is the number of C-atoms and N-atoms in the cation developed using the data in this table. The value in brackets was not taken into correlation.

[b] Difference between column 3 and 4.

[c] The heat capacities differences were derived from an empiric equation:

$\Delta C^{o}_{p,m}$ =-0.126×$C^{o}_{p,m}(liq)_{exp}$ – 1.5 (with R^2 = 0.987) (see Table S4). The heat capacities values $C^{o}_{p,m}(liq)_{exp}$ used were taken from this table.

Table S4

Correlation of molar heat capacities, $C^{o}_{p,m}(liq)$, and molar heat capacities differences $\Delta^{g}_{l}C^{o}_{p,m}$ (in J·K^{-1}·mol^{-1}) at 298.15 K

	$C^{o}_{p,m}(liq)_{exp}$ [a]	$-\Delta^{g}_{l}C^{o}_{p,m}$ [b]	$-\Delta^{g}_{l}C^{o}_{p,m}(calc)$ [c]
[1-C_2-Py][NTf_2]	478.6	61	62
[1-C_3-Py][NTf_2]	503.4	66	65
[1-C_4-Py][NTf_2]	540.2	70	70
[1-C_5-Py][NTf_2]	569.3	73	73
[1-C_6-Py][NTf_2]	599.5	77	77

[a] From Table S3.

[b] The $\Delta^{g}_{l}C^{o}_{p,m}$–values derived in our previous work [7] using a procedure based on the volumetric properties of ionic liquids.

[c] Calculated using correlation: $\Delta C^{o}_{p,m}$ =-0.126×$C^{o}_{p,m}(liq)_{exp}$ – 1.5 (with R^2 = 0.987), derived from data given in this table. This correlation was propagated to the alkyl- and cyano-substituted pyridinium based ILs connected with the [NTf_2]-anion.

Table S5

Calculation of vaporization enthalpies, $\Delta_l^g H_m^o$, of alkyl- and cyano-substituted pyridinium and based ILs using the "centerpiece approach" (at 298.15 K in kJ·mol^{-1})[a]

IL	$\Delta_l^g H_m^o$(**H→R₁**)[b]	$\Delta_l^g H_m^o$(CP)[c]	Correction[d]	$\Delta_l^g H_{m(add)}^o$[e]
[3-Me-1-C_2-Py][NTf_2]	4.4±0.3	131.7±1.4	-4.9±0.8	131.2±1.6
[2-Et-1-C_2-Py][NTf_2]	5.7±0.2	131.7±1.4	-4.9±0.8	132.5±1.6
[3-Me-1-C_3-Py][NTf_2]	4.4±0.3	135.4±1.5	-4.9±0.8	134.9±1.7
[4-Me-1-C_3-Py][NTf_2]	4.7±0.3	135.4±1.5	-4.9±0.8	135.2±1.7
[4-Me-1-C_4-Py][NTf_2]	4.7±0.3	137.8±1.4	-4.9±0.8	137.6±1.6

[a] Uncertainties of vaporization enthalpy ($\Delta_l^g H_m^o$) are the expanded uncertainties (0.95 level of confidence, k = 2).
[b] From Table 9.
[c] Enthalpies of vaporization of the "centerpiece" molecules from Tables 1 - 3.
[d] Correction from Table 11.
[e] Calculated as the sum of columns 2+3+4.

Gas-Chromatographic method (GC): The activity coefficient at infinite dilution γ_1^∞ of a volatile solute (typically an industrially relevant solvent such as benzene, methanol, acetone, *etc*., denoted with a subscript (1) in an ionic liquid (denoted with a subscript 2) are in excess represented in the literature. The γ_1^∞-values are conveniently measured by using gas-chromatography [8]. Usually, a set of γ_1^∞-values for 20-40 solutes of different polarity is reported for a particular IL.

The γ_1^∞ values are related to the total solubility parameter δ_1 for a solute and δ_2 for an IL. In turn, the total solubility parameter (δ_T) at a temperature T is related to vaporization enthalpy over equation [9]:

$$\delta_T = [(\Delta_l^g H_m^o - RT)/V_m]^{0.5} \quad \text{(S4)}$$

where $\Delta_l^g H_m^o$ is the standard molar enthalpy of vaporization (of solute or solvent), R is the ideal gas constant, T is the temperature, and V_m is the molar volume (of solute or solvent). Solubility parameter δ_1 and δ_2 are responsible for mutual miscibility of a solute and an IL. Quantitatively it is represented over a Flory–Huggins interaction parameters, χ_{12} at infinite dilution [10]:

$$x_{12} = \frac{V_1^*(\delta_1 - \delta_2)^2}{RT} \quad \text{(S5)}$$

where δ_2 is the solubility parameter of the particular IL and δ_1 is the solubility parameter of the solute of interest, R denotes the universal gas constant, T is the temperature, V_1^* is the molar volume of the solute. After a simple algebraic rearrangement of Eq. S5 it gives the following equation [10]:

$$\frac{\delta_1^2}{RT} - \frac{\chi_{12}}{V_1^*} = \left(\frac{2\delta_2}{RT}\right)\delta_1 - \frac{\delta_2^2}{RT} \quad \text{(S6)}$$

The Flory–Huggins interaction parameter χ_{12} at infinite dilution can be derived from experimental activity coefficient at infinite dilution γ_1^∞ according to Eq. S7:

$$x_{12} = \ln\left(\frac{273.15\,\gamma_1^\infty M_2}{TM_1}\right) - \left(1 - \frac{V_1^*}{V_2^*}\right) + \ln\left(\frac{\rho_1}{\rho_2}\right) \quad \text{(S7)}$$

where M_1 and M_2 are the molecular weight of solute and solvent, V_1^* and V_2^* and ρ_1 and ρ_2 are the molar volume and density of solute and solvent, respectively. From a graphical representation of Eq. S6 (Fig. S6), it is apparent: when the left side of Eq. S6 is plotted against δ_1, the expression $2\delta_2/(RT)$ is the slope of the line and the expression $-\delta_2^2/(RT)$ is the intercept. Using linear regression of the experimental γ_1^∞-values, the slope and intercept can be used to determine the solubility parameter of an ionic liquid δ_2. Consequently, the vaporization enthalpy, $\Delta_l^g H_m^o$(298.15 K) of an IL under study can be calculated using Eq. S4 and the δ_2-value as follows:

$$\Delta_l^g H_m^o(T) = [\delta_2^2 \times V_m + RT] \quad \text{(S8)}$$

where all values, including V_m are referenced to an arbitrary temperature T, which is 298.15 K in this work.

We fitted Eq. S6 with γ_1^{∞}-values for [4-Me-C4-Py][NTf2] measured by Domańska and Marciniak [11]. Table S7 identifies the solutes and the solvents used to obtain δ_2. The δ_2-values were estimated as the average obtained from the slope and the intercept of Eq. S6. Values $\Delta_l^g H_m^o$(298.15 K) were calculated from δ_2 according to Eq. S8. The result $\Delta_l^g H_m^o$(298.15 K) = 135.7±3.0 kJ·mol^{-1} (see Table S6) is in good agreement with the "centerpiece" calculated value $\Delta_l^g H_m^o$(298.15 K) = 137.6±1.6 kJ·mol^{-1}, giving independent support for evaluation performed in Table S5.

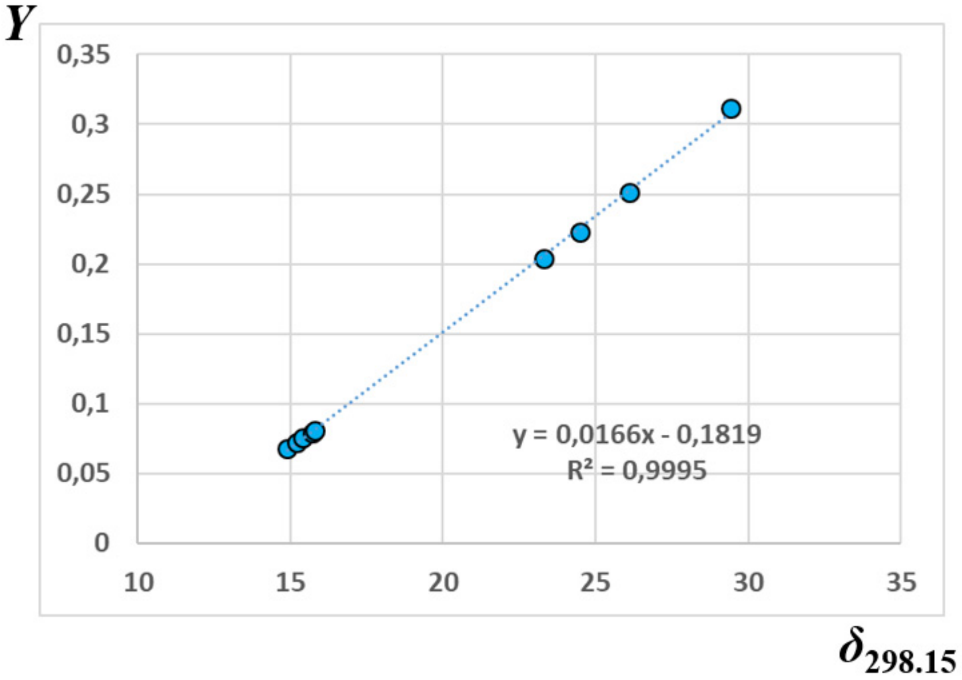

Figure. S6 Regression of solubility parameters $\delta_{298.15}$ of different solutes and ***Y***-module (***Y*** = $(\delta_1)^2/(RT)$ - χ_{12}/V_1^* is the left part of Eq. S6) for [4-Me-1-C4-Py][NTf2] derived from experimental γ_1^{∞}-values [11].

Table S6

Results of regression with Eq. (S6) for [4-Me-1-C4-Py][NTf2]

		δ_2	
slope	0.0166	20.6	
intercept	-0.1820	21.2	
average		20.9	$\Delta_l^g H_m^o$(298.15 K) = 135.7±3.0 kJ·mol^{-1}

Table S7. Data used for regression with Eq. (S6) for [4-Me-C_4-Py][NTf_2]: solubility parameters $\boldsymbol{\delta_{298.15\,K}}$ of different solutes in at 298.15 K and $\boldsymbol{Y}$ - the left part of Eq. (S6), derived from experimental γ_1^∞-values [11]

solute	$\boldsymbol{\delta_{298.15\,K}}$ /MPa$^{0.5}$	$\boldsymbol{Y}^{a}$
heptane	15.2	0.072
octane	15.4	0.0755
nonane	15.7	0.079
decane	15.8	0.0804
methanol	29.4	0.3115
ethanol	26.1	0.2513
1-propanol	24.5	0.223
1-butanol	23.3	0.2039

[a]The left part of Eq. (S6): $\boldsymbol{Y} = (\delta_1)^2/(RT) - \chi_{12}/V_1{}^*$

Table S8

Correlation of vaporization enthalpies, $\Delta_l^g H_m^o$(298.15 K), of [1-C_n-Py][NTf_2] series with the chain length [a]

Compound	n [b]	$\Delta_l^g H_m^o$(298 K)$_{exp}$[c] kJ·mol^{-1}	$\Delta_l^g H_m^o$(298 K)$_{calc}$[c] kJ·mol^{-1}	Δ[d] kJ·mol^{-1}
[1-C_2-Py][NTf_2]	2	131.7	131.4	0.3
[1-C_3-Py][NTf_2]	3	135.4	135.0	0.4
[1-C_4-Py][NTf_2]	4	137.8	138.6	-0.8
[1-C_5-Py][NTf_2]	5	141.7	142.2	-0.5
[1-C_6-Py][NTf_2]	6	146.1	145.8	0.3
[1-C_7-Py][NTf_2]	7	-	149.4	-
[1-C_8-Py][NTf_2]	8	153.2	153.0	0.2
[1-C_9-Py][NTf_2]	9	-	156.6	-
[1-C_{10}-Py][NTf_2]	10	-	160.2	-

[a] Uncertainties in this table are expressed as the twice standard deviation.

[b] The number of C-atoms in the alkyl-chain attached to the nitrogen of the cation.

[c] From Table 2, main text.

[d] Calculated using equation: $\Delta_l^g H_m^o$(298.15 K) /(kJ·mol^{-1}) = 124.2 + 3.60×n with (R^2 = 0.9958) and with the assessed uncertainty of ±2.0 kJ·mol^{-1}.

[e] Difference between column 3 and 4 in this table.
